# How does facilitation in healthcare work? Using mechanism mapping to illuminate the black box of a meta-implementation strategy

**DOI:** 10.1186/s43058-023-00435-1

**Published:** 2023-05-16

**Authors:** Amy M. Kilbourne, Elvin Geng, Ingrid Eshun-Wilson, Shannon Sweeney, Donna Shelley, Deborah J. Cohen, JoAnn E. Kirchner, Maria E. Fernandez, Michael L. Parchman

**Affiliations:** 1 Health Services Research & Development, VA Office of Research and Development, US Department of Veterans Affairs and University of Michigan, 810 Vermont Ave, NW, Washington, D.C., 20420 USA; 2 Washington University at St. Louis, St. Louis, MO USA; 3 Oregon Health & Science University, Portland, OR USA; 4 New York University School of Global Public Health, New York, New York USA; 5 Central Arkansas VA Healthcare System and University of Arkansas for Medical Sciences, North Little Rock, AR USA; 6 University of Texas Health Science Center at Houston, School of Public Health, Houston, TX USA; 7 Kaiser Permanente Washington Health Research Institute, Seattle, WA USA

**Keywords:** Facilitation, Mechanism mapping, Implementation strategies, Quality improvement

## Abstract

**Background:**

Healthcare facilitation, an implementation strategy designed to improve the uptake of effective clinical innovations in routine practice, has produced promising yet mixed results in randomized implementation trials and has not been fully researched across different contexts.

**Objective:**

Using mechanism mapping, which applies directed acyclic graphs that decompose an effect of interest into hypothesized causal steps and mechanisms, we propose a more concrete description of how healthcare facilitation works to inform its further study as a meta-implementation strategy.

**Methods:**

Using a modified Delphi consensus process, co-authors developed the mechanistic map based on a three-step process. First, they developed an initial logic model by collectively reviewing the literature and identifying the most relevant studies of healthcare facilitation components and mechanisms to date. Second, they applied the logic model to write vignettes describing how facilitation worked (or did not) based on recent empirical trials that were selected via consensus for inclusion and diversity in contextual settings (US, international sites). Finally, the mechanistic map was created based on the collective findings from the vignettes.

**Findings:**

Theory-based healthcare facilitation components informing the mechanistic map included staff engagement, role clarification, coalition-building through peer experiences and identifying champions, capacity-building through problem solving barriers, and organizational ownership of the implementation process. Across the vignettes, engagement of leaders and practitioners led to increased socialization of the facilitator’s role in the organization. This in turn led to clarifying of roles and responsibilities among practitioners and identifying peer experiences led to increased coherence and sense-making of the value of adopting effective innovations. Increased trust develops across leadership and practitioners through expanded capacity in adoption of the effective innovation by identifying opportunities that mitigated barriers to practice change. Finally, these mechanisms led to eventual normalization and ownership of the effective innovation and healthcare facilitation process.

**Impact:**

Mapping methodology provides a novel perspective of mechanisms of healthcare facilitation, notably how sensemaking, trust, and normalization contribute to quality improvement. This method may also enable more efficient and impactful hypothesis-testing and application of complex implementation strategies, with high relevance for lower-resourced settings, to inform effective innovation uptake.

**Supplementary Information:**

The online version contains supplementary material available at 10.1186/s43058-023-00435-1.

Contributions to the literature

- Healthcare facilitation is an increasingly popular implementation strategy, yet empirical studies of its effectiveness have lacked deep analysis of its mechanisms.
- Mechanism mapping is a promising approach to help determine how facilitation works in different contexts.
- Across different contexts, healthcare facilitation is potentially effective because of its hypothesized impacts on organizational sense-making, provider trust, and normalization of a practice change, informing its future exploration and practice as an implementation strategy.

## Introduction

Facilitation in healthcare settings (also referred to as practice facilitation or implementation facilitation, and henceforth referred to as healthcare facilitation) is an implementation strategy [1, 2] that supports people in health services organizations develop the means to change the structure and processes within settings to help reduce the gap between evidence and practice [3–7]. Healthcare facilitation has been described as a cyclical, dynamic learning process in which facilitators apply diverse strategies through interactive problem-solving in a supportive relationship among healthcare employees that may lead to performance improvement [8–24].

The major theories and frameworks that have informed healthcare facilitation components include organizational learning theory [4], normalization process theory [25], health systems frameworks, notably the Integrated Promoting Action on Research Implementation in Health Services [7], and complexity science [26–29]. These theories and frameworks are also essential to understanding the context in which healthcare facilitation is effective, notably capacity, culture, climate, or policies [23, 30–34] as well as mediators (e.g., provider self-efficacy, burnout, staffing turnover, leadership perceptions) in industrialized [35, 36] as well as in low- and middle-income countries [37–40].

However, evidence about whether healthcare facilitation is effective in improving clinical processes (e.g., provider uptake of effective innovations) and/or improved patient outcomes has been mixed [3, 6, 9, 10, 14, 16, 19–24, 41–49], especially in situations when facilitation was delivered virtually or in low- and middle-income countries; see Additional file 1 for a summary of recent healthcare facilitation trials. Key reasons include inconsistent approaches and assessment of healthcare facilitation driven by limited research on its core components and mechanisms, that is, what makes facilitation work, and how it works to improve adoption and implementation. Limited descriptions of its core components, mechanisms, and underlying contextual factors preclude the ability to apply facilitation consistently and test hypotheses derived from theories and frameworks to advance our understanding of how healthcare facilitation works [7–11]. Understanding the core components of healthcare facilitation and specifying the mechanisms by which facilitation improves clinical processes and outcomes, may improve the study and delivery of this implementation strategy.

The goal of this paper is to apply a novel method, mechanism mapping, to provide a more concrete description regarding how healthcare facilitation works that can be applied and tested in future studies. A relatively new method to implementation science that has been previously used in policy analysis, mechanism mapping [50], can help with identifying the causal relationships, either observed or hypothesized, that depict how the implementation strategy achieves its effect in practice change and/or outcomes improvement. To date, mechanism mapping has not been applied to specific implementation strategies, notably healthcare facilitation, despite the substantial number of studies using this implementation strategy and its increasing popularity with funding agencies and health systems such as the U.S. Agency for Healthcare Research and Quality and Department of Veterans Affairs. In doing so, the authors draw upon theory, frameworks, and previous empirical findings to first identify core components of healthcare facilitation. Using mechanism mapping, these components are then aligned in causal order based on the experiences of recent multisite studies testing healthcare facilitation as an implementation strategy with a particular focus on lower-resourced US care settings as well as low- and middle-income countries.

## Methods

The healthcare facilitation mechanistic map was developed using a modified Delphi consensus process [51] based on a previously established implementation science-focused consensus process [52]. Nine experts in healthcare facilitation research and application met virtually five times between October 2021 and May 2022 to develop the healthcare facilitation mechanistic map via active discussions that applied literature synthesis, logic modeling, and vignette development from empirical studies to identify and map healthcare facilitation components and processes. Participants (all co-authors) are experts in the development and application of healthcare facilitation across different contexts. Meeting notes were recorded by study co-authors using Zoom.

### Procedures

The group used a three-stage process to develop the mechanistic map for healthcare facilitation. First, group members identified and reviewed key papers on healthcare facilitation theory, frameworks, and evidence reviews (summarized in Additional file 1) to develop a healthcare facilitation logic model. Second, the group wrote vignettes based on their experiences from recent empirical trials to better describe mechanisms and contextual factors of healthcare facilitation. Finally, the mechanistic map was developed from these vignettes.

### Logic model

The group developed a logic model of healthcare facilitation that was informed by the Implementation Research Logic Model [53]. Specifically, the logic model was designed to help specify the components and potential mechanisms of healthcare facilitation in order to enable reproducibility and testing of causal pathways in a linear fashion. This logic model was used initially because it described a primarily linear process of an implementation strategy whereas the mechanism mapping process is acyclic, which enables description of how an implementation strategy might work in cycles or phases from treatment to outcome. The healthcare facilitation logic model was informed by a literature review of known and hypothesized mechanisms and contextual factors affecting healthcare facilitation from the literature as well as collective experience in training and using healthcare facilitation in implementation trials. The group first synthesized current knowledge of healthcare facilitation components and proposed mechanisms. Findings from this review, summarized in Additional file 1, focused on more recent empirical studies of healthcare facilitation, given prior systematic reviews of healthcare facilitation [43, 45]. The literature review was based on co-author consensus via group discussions that identified the most relevant articles describing facilitation components as well as recent empirical studies that helped to elucidate its mechanisms. Additional file 1 also includes a summary of the core components of healthcare facilitation based on the literature review using the Proctor Actor-Action framework [1]. The logic model was reviewed iteratively and refined based on the group’s experiences with research findings on how contextual factors interact with and may modify the effect of healthcare facilitation and the proposed components and mechanisms that might influence practice change, effect innovation uptake, and improve clinical outcomes.

### Vignettes

Group members each developed vignettes based on purposeful sampling of recent implementation studies using healthcare facilitation to ensure representation across different organizational contexts (e.g., integrated health systems, small practices in industrialized countries, low- and middle-income countries). Vignettes that derived from studies involving at least one of the co-authors who could provide more in-depth information on the study methods beyond the published papers were given preference for selection. A total of four vignettes were included after a consensus process where each vignette represented a different contextual setting (e.g., government-managed, specialty care, small primary care practice, and low- and middle-income country settings). For each vignette, the group member involved in the study provided a brief description of the study goal, setting, and findings including impact of healthcare facilitation on clinical processes and/or patient outcomes, as well as mechanisms where relevant. After writing the summary, the group reviewed each vignette to detail how the study’s findings informed the mechanistic map.

### Mechanism mapping

The mechanistic map for healthcare facilitation was developed using directed acyclic graphs to decompose an effect of interest into component causal steps, from “treatments” (i.e., causes such as implementation strategies), to “outcomes” (i.e., effects, such as practice change), and mediators of these effects. Mechanistic maps share similar features with intervention mapping [54] yet attempt to delineate in a more non-linear fashion the overall mechanisms of an implementation strategy such as healthcare facilitation. These representations of hypothesized effects are not easily captured in either statistical notation nor in language, and therefore creation of the diagram helps investigators develop a shared understanding of how an effect of interest occurs, the major pathways, how these pathways interact with each other, and how contextual factors influence one or more mechanisms. The directed acyclic graph assumes that any “path” started at a particular node may not return to that node at any point. It is also possible for the graphs can decompose feedback loops into their discrete temporal stages and represent them as time-dependent or time-varying confounding [55]. This is critical for their application to healthcare facilitation given that there is a preponderance of evidence that healthcare delivery settings are complex adaptive systems, characterized by non-linear interactions and emergent properties [56, 57].

In the mechanism mapping approach, we apply the directed acyclic graphs method to encode hypothesized relationships between key elements of healthcare facilitation and use this approach to dissect how these complex activities are believed to work. The proposed relationships in the mechanistic map were developed via consensus by the co-authors based on a review of the recent literature, construction of a healthcare facilitation logic model, and development of vignettes describing how facilitation worked (or did not) across different contexts. Proposed mechanisms were further elaborated based on the four vignettes derived from implementation studies conducted in different healthcare settings that illustrate examples of healthcare facilitation in practice. For each vignette, study co-authors walked through observed healthcare facilitation mechanisms identified in their research studies and proposed causal links between mechanisms. After consensus, the causal links common to these vignettes are then represented in the mechanistic map.

## Results

### Logic model results

**Fig. 1:**
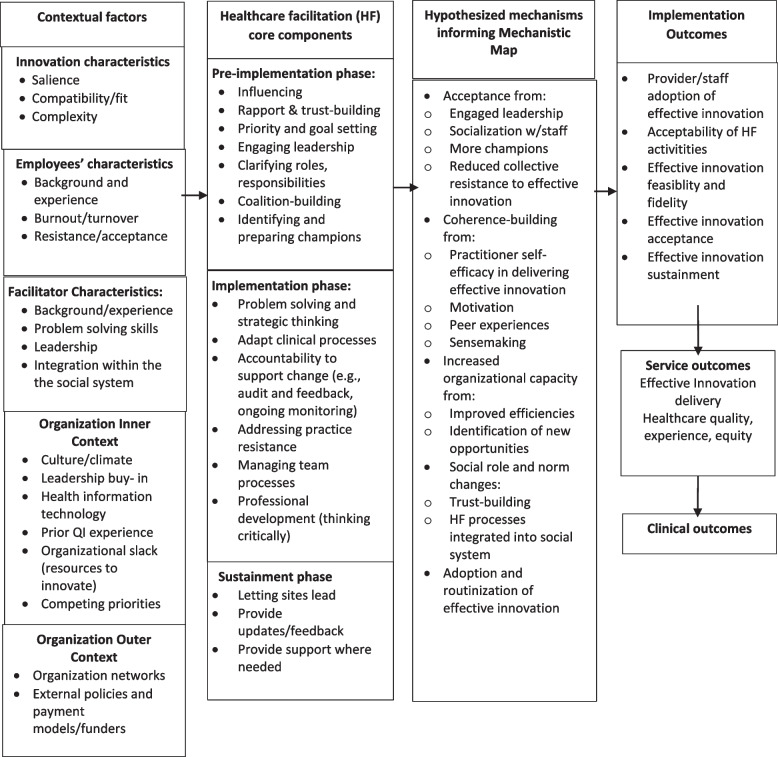
Healthcare facilitation logic model

The healthcare facilitation logic model (Fig. 1) outlines the components to be included in the mechanistic map. As bookends, the logic model identifies the underlying contextual factors (outer setting, inner setting) and desired outcomes (e.g., implementation, uptake, patient outcomes) derived from the literature and recent studies (see Additional file 1). The implementation actions described in the Additional file 1 table represent key implementation outcomes of healthcare facilitation from the recent empirical studies reviewed [3, 6, 9, 12, 16, 19, 20, 23, 37–40, 45–49].

Core components delivered by the healthcare facilitator are depicted in the second set of columns of the logic model and are primarily derived from empirical studies of healthcare facilitation. Core components included the following: (1) engagement of practitioners through priority and goal setting, (2) clarifying roles and responsibilities, (3) coalition-building across leaders and champions to help build organizational capacity for the effective innovation, (4) continuous problem-solving, strategic thinking, and adaptation, and (5) integration of innovation and facilitation components into the organization and letting sites lead the implementation.

**Fig. 2:**
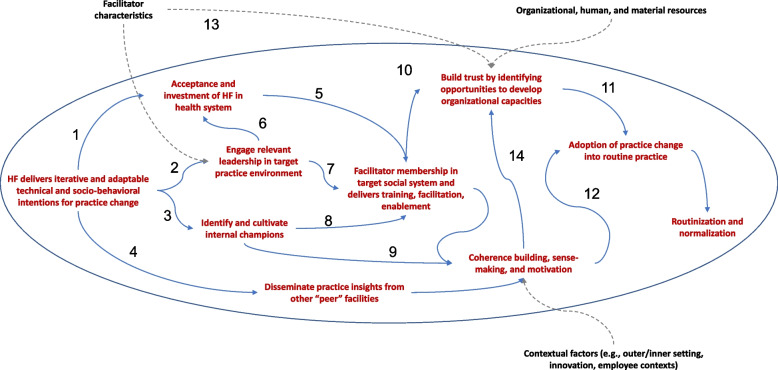
Healthcare facilitation (HF) mechanism map using directed acyclic graphs

Proposed hypothesized mechanisms in the logic model based on theory and empirical research were then listed in the third column but presented in a linear fashion. Key mechanisms (e.g., acceptance, coherence-building, increased organizational capacity, social role change, and adoption/normalization of practice change) were then further elaborated in the acyclic mechanism mapping in Fig. 2. For this reason, the results from the mechanistic map are presented first, followed by the vignette descriptions that informed the map.

### Mechanism mapping results

In applying mechanism mapping to healthcare facilitation (Fig. 2), we suggest that healthcare facilitation is a planned process in which an actor (facilitator) seeks to create bonds with the social system of an organization by delivering socio-technical skills and processes to enhance practice change through effective innovations. The healthcare facilitator’s activities are planned, iterative, and adaptive. That is to say the exact intensity and nature of interaction will depend on how the organization responds.

Initially, the healthcare facilitator engages and achieves acceptance by the involved social system. This is a complex process that involves relationship-building through interpersonal connections among the practitioners in the organization and can be influenced by the characteristics and skills of the facilitator. Healthcare facilitators may also engage leadership in the organization because leadership investment can (1) improve the facilitator’s ability to gain traction in a new social system, (2) promote broader use of technical skills among staff, and (3) identify and prepare other champions for innovation. Engagement with healthcare leadership allows the healthcare facilitator to catalyze a series of changes that involve coherence and coordination around implementing an effective innovation and the subsequent development of organizational capacities supporting the innovation. However, the temporal aspect of this work introduces time-varying confounders that may result in non-linear interactions. For example, the facilitator may influence and also be influenced by leadership. As leadership observes a facilitator gaining traction in their local social system, this may enhance leadership engagement in their work. This is a time-dependent phenomenon and as such it can be represented as a confounder in mechanism mapping diagrams.

### Facilitation vignettes

The vignettes described below demonstrate how these healthcare facilitation mechanisms in Fig. 2 play out across different contexts. In these situations, healthcare facilitation was delivered directly to providers at the practice level.

### Healthcare facilitation in a national government-based health system (Veterans Health Administration, VHA)

Bauer and colleagues assessed the impact of healthcare facilitation on the uptake of an evidence-based collaborative care model and patient outcomes [58–62] in nine outpatient VHA general mental health clinics [60, 61]. Sites were recruited through the operational partner, the VHA Office of Mental Health and Suicide Prevention, thus engaging partners in the use of the practice (Arrow #6, Fig. 2). Each site identified a staff member to serve as an internal healthcare facilitator with time protected by the site for implementation activities (Arrow #8, Fig. 2). Healthcare facilitation activities included an assessment of determinants that could impact successful implementation, orientation of the site leadership and staff to healthcare facilitation activities, a face-to-face site visit to launch the implementation process, and weekly virtual meetings for six months to audit and support implementation (Arrows #1, #10, Fig. 2). In addition, 6 months of step-down facilitation was provided on an as needed basis (Arrow #12, Fig. 2). Findings from this body of work resulted in the operational partner’s scale-up and spread to additional medical centers [63] allowing for the adoption of the collaborative care model into routine clinical practice as well as tools to promote ongoing facilitation support [64, 65] (See Arrows #4, #11, Fig. 2). However, in the year following the cessation of active implementation support [66], effects on hospitalization rate became non-significant, suggesting that more attention might need to be paid to sustainment.

### Healthcare facilitation in community mental health practices

The Adaptive Implementation of Effective Programs Trial [22, 23, 34, 63] compared the effectiveness of an external versus external + internal facilitator on the uptake of an intervention based on the collaborative care model, Life Goals Collaborative Care, among 58 community mental health and primary care clinics using a sequential multiple assignment randomized trial design [67, 68]. While the study team hired and trained external facilitators, sites randomized to receive internal facilitation were asked to identify an additional internal facilitator (e.g., clinic manager) whose effort was supported by the study. Sites receiving external facilitation experienced greater improvements in patient outcomes (quality of life, symptom reduction) [23] and Life Goals Collaborative Care uptake [34] than sites that received external + internal facilitation. By serving as outside experts, external facilitators may have built more trust among leaders and staff (Arrow #13, Fig. 2) and subsequently were able to enhance capacity for collaborative care model implementation [34]. This was enabled through initial sense-making (Arrows #2, #7, #5, Fig. 2) where external facilitators were able to engage with leadership to identify opportunities for alignment with organizational goals and to offer continuous training (Arrow #10, Fig. 2). In contrast, having leadership identify an appropriate internal healthcare facilitator to further embed Life Goals Collaborative Care was challenging due to the internal facilitators’ lack of time. Moreover, some may have felt “volun-told” to participate (Arrow #11, Fig. 2). What was less successful, and hence, led to limited program sustainment after the study, was the ability for the external or internal facilitators to identify and prepare practice team-level champions to continue the normalization and adoption of Life Goals Collaborative Care as part of routine care (Arrows #9 and #12, Fig. 2).

### Healthcare facilitation in small practices and federally qualified health centers

As part of the U.S. Agency for Healthcare Research and Quality-funded EvidenceNOW initiative [69], 15 healthcare facilitators worked with publicly funded practices in New York City to implement the Million Hearts guidelines for preventing and treating cardiovascular disease [31]. Healthcare facilitators addressed contextual factors that map to the inner and outer organizational contexts represented in Fig. 1, notably policy and payment environment, organizational capacity and competing priorities (e.g., clinician time and resources to adopt new quality improvement initiatives), and leadership engagement [31]. Due to staffing shortages and competing priorities among existing staff, healthcare facilitators adopted additional roles such as training and coaching to help with specific skills and hence became part of the practice social system (Arrows #1 and #7, Fig. 2). Strategies mitigating contextual barriers included sense-making, such as remaining flexible to align with practice and organizational priorities (Arrow #5, Fig. 2), creating norms through cross conversations and networking opportunities with other practices (Arrow #4, Fig. 2), providing value through sharing information technology expertise, and building capacity and creating efficiencies (Arrow #11, Fig. 2). As represented by Arrow #13 in Fig. 2, facilitators were able to build trust by identifying new opportunities to further promote the organization’s capacity to implement. Addressing competing priorities (i.e., organizational context) by creating efficiencies through workflow redesign (Arrow #11, Fig. 2) was particularly important in small practices with few staff to help reduce or redistribute their workload. Within the different practice contexts understanding the practice assets, constraints and priorities informed opportunities including necessary adaptations to these approaches. Remaining flexible was an example of a tailoring strategy in which opportunities were identified to further adapt the effective innovation to different settings (Arrows #10, #11, Fig. 2).

### Healthcare facilitation in networked public health facilities in low- and middle-income countries

The Patient Centered-Care study [70] was a stepped-wedge randomized controlled trial across 24 primary and secondary public health facilities in Lusaka Province, Zambia, that used a healthcare facilitation strategy to promote patient-centeredness of HIV care, with the goal of improving patient experience and clinical and care outcomes. Current findings suggest that healthcare facilitators highlighted the critical importance of leadership buy-in through extensive preparatory work that engaged facility leadership (Arrows #1 and #6, Fig. 2). Leadership buy-in markedly influenced the dose and intensity of facilitation permitted and the receptiveness of health workers to healthcare facilitation. Moreover, ongoing dialogue between healthcare facilitators led to problem-solving to overcome barriers to uptake and improve overall implementation throughout the facilitation period (Arrows #5 and #10, Fig. 2). Early identification of internal facilitators who were engaged in the implementation process was also critical for successful implementation (Arrow #3, Fig. 2). The dose and intensity of facilitation were titrated to the facility needs with high initial intensity required during early stages and tapering during later stages when the Patient Centered Care study principles were more routinely adopted (Arrow #11, Fig. 2). Feedback from facilitators identified the benefits of ensuring that facilitators are sufficiently networked to increase learning and support (Arrow #4, Fig. 2). Facilitators also highlighted that relationships with facility staff were strengthened by embedding themselves within clinic operations, task sharing and assisting with routine clinic work during busy periods (Arrows #5 and #15, Fig. 2).

### Summary

Overall, the vignettes depict a common set of healthcare facilitation mechanisms that are outlined in the logic model (Fig. 1, column 3) and further mapped out in the acyclic graph (Fig. 2). The mechanistic map also suggests potential dependencies of the steps, multiple pathways, and how pathways interact. By decomposing time-dependent confounders, the mechanistic map also represents inherent non-linearity of healthcare facilitation in a complex system, such as what is often observed by healthcare facilitators who have experienced this first-hand [8, 46].

The mechanistic map may also elucidate candidate hypotheses regarding the causal pathways of healthcare facilitation. The following description of such a pathway is based on the experiences observed across the vignettes. First, engagement of leaders and practitioners led to increased socialization of the facilitator’s role in the organization. Second, clarifying roles and responsibilities among practitioners and identifying peer experiences led to increased coherence and sense-making of the value of adopting effective innovations. Third, increased trust occurred across leadership and practitioners through expanded capacity in adoption of the effective innovation by mitigating barriers to practice change. Finally, these mechanisms led to eventual normalization and ownership of the effective innovation and healthcare facilitation process.

## Discussion

Using mechanism mapping, applied acyclic graphs were used to further elucidate the components and key mechanisms of healthcare facilitation. These mechanisms were identified using findings from a logic model derived from underlying theory and frameworks and applying vignettes from empirical research. Mechanism mapping elucidated potentially new pathways in socialization, sense-making, trust building, and normalization within the organization by which healthcare facilitation might work that can be further tested in empirical trials. Of note, acceptance of the innovation across different groups within each of the vignettes potentially led to sense-making among care teams, creative resourcing that improved efficiencies, and eventually, social norm, role changes within organizations, and sustainment through trust-building and normalization of the innovation in practice.

To date, previous implementation studies rarely described the mechanisms of healthcare facilitation and, if so, were not presented as a dynamic process. In addition, healthcare facilitation components often lacked a standard terminology across studies [14]. Healthcare facilitation mechanisms have also been difficult to elucidate previously because of the changing healthcare landscape and the dynamic process by which quality improvement occurs that preclude a more linear, fixed logic or mechanism-based model. Practice context can also significantly influence the success of healthcare facilitators and how they can adapt their support to respond to a range of barriers.

Mechanism mapping using acyclic graphs is an innovative methodology that can potentially advance our understanding of how and why healthcare facilitation works to improve implementation of effective innovations. Notably, the use of acyclic graphs allows for hypothesized mechanisms to include both cause and effect of factors such as leadership support and higher-level organizational learning. This approach also builds upon complementary methods previously used to describe how healthcare facilitation and other implementation strategies work in practice including configurational analysis [71, 72], matrixed multiple case study analysis [73], and other emerging tools and checklists [74, 75].

As it gains popularity in US and international settings, understanding the phenomenon of healthcare facilitation, especially when it fails to achieve desired outcomes, is essential to ensure its consistent and high-quality application to improve uptake of effective innovations and quality/outcomes of care. Emerging scientific mechanism methodologies are also needed to better capture the details of each component of healthcare facilitation, how they are adapted in different contexts and why. Mechanism mapping using acyclic graphs can help overcome common challenges to studying healthcare facilitation, notably by focusing on common terminologies for actions and identifying core contextual characteristics that influence its effectiveness.

Despite the novel application of mechanism mapping to an implementation strategy, there are limitations to this study that warrant consideration. The rapidly evolving nature of healthcare facilitation studies precluded a comprehensive review of its effectiveness. Moreover, the current healthcare facilitation examples also varied in their approach to documenting mechanisms quantitatively. Mechanism mapping may not capture the full array of complex relationships evidenced in healthcare organizations or may lead to perceptions that the relationships are more linear than what is experienced in real-world settings. As complex adaptive systems, healthcare organizations have many inputs that might not be observable and therefore measured to be included in mechanism mapping. Many of the steps in the mechanism mapping are context-dependent (e.g., adoption of routine practices largely depended on buy-in from practitioners). The vignettes and studies used to inform the mechanism mapping were not all based on randomized designs, thereby contextual factors could be predictive of both the facilitation activities undertaken and the ultimate success of the study. Still, the mechanism mapping attempts to set boundaries for where these mechanisms might exist in the array of actions across time and space in healthcare settings. Or, in other situations, some of the mechanisms remain dormant until activated by a sense of urgency (e.g., such as the potential ending of a study that provides healthcare facilitation support to sites). In addition, the process of identifying and mapping core components of healthcare facilitation may have missed key elements not observed in the vignettes, and the mechanism mapping results may not be generalizable beyond lower-resourced US or low- and middle-income country settings. However, further application of mechanism mapping to more diverse settings may shed additional light on the content and process by which this meta-implementation strategy is effective, especially across international settings.

Moreover, given rapid changes in healthcare resources especially with the recent pandemic (e.g., staffing and supply shortages, move to virtual care), a more nuanced, cyclical approach is needed to explain the dynamic process of practice and provider engagement in healthcare facilitation activities over time. Understanding which components work best and why will enable more efficient use of healthcare facilitation in lower-resourced settings. Perhaps the use of acyclic graphs and hypothesis testing about mechanisms of facilitation are best accomplished in real-world care contexts with study designs that have the potential to improve care. Further research on the use of mechanism mapping to describe healthcare facilitation and other implementation strategies might also benefit from emerging technology-focused methods including user-centered design [76] as well as machine learning, especially if process and quality improvement data can be derived from electronic health records [77].

## Conclusions

Healthcare facilitation is a complex process but with mechanism mapping its key elements are further elucidated. Additional research on how healthcare facilitation leads to socialization, sense-making, trust building, and normalization in organizations to support practice change is needed to further improve this increasingly used implementation strategy. In addition, mechanism mapping can inform our understanding of how healthcare facilitation succeeds or fails when applied in a specific setting and can be used to test hypotheses derived from existing theories to develop a formal model of anticipated mechanisms. Overall, as healthcare evolves in the USA and globally to meet the needs of changing health systems and emerging public health issues, there will be a greater demand for a more tailored and nuanced approaches to practice change particularly as providers are being asked to do more in an increasingly complex healthcare system. Healthcare facilitation and the use of mechanism mapping to elucidate its active ingredients have great potential to support practitioners and their healthcare teams face a changing world with resilience, flexibility, rigor, and understanding.

## Supplementary Information

**Additional file 1.** Summary.

## Data Availability

N/A
