# Supplementary material for: How does facilitation in healthcare work? Using mechanism mapping to illuminate the black box of a meta-implementation strategy
Source: Implement Sci Commun. 2023 May 16;4:53. doi: 10.1186/s43058-023-00435-1 (PMC10190070; doi:10.1186/s43058-023-00435-1)
Supplement: Supplementary file 1 — Additional file 1. Summary. [file 43058_2023_435_MOESM1_ESM.docx]

**Additional background on healthcare facilitation theories, frameworks, and evidence**

This section describes background information that was accumulated to inform a process for creating a mechanistic map of healthcare facilitation. While not an exhaustive review of the literature, this summary highlights the core theories and frameworks that have been used to inform the core components of facilitation, as well as a summary of the current literature on the effects of healthcare facilitation on uptake and quality of care. This summary informed an initial description of healthcare facilitation using the Proctor framework (see Table below) that was used by the study’s modified Delphi panel to inform the logic model and ultimately, the mechanistic map of this meta-implementation strategy.

**Healthcare facilitation underlying theory and frameworks**

The major theories and frameworks that have informed healthcare facilitation components and more recently, mechanisms, include organizational learning theory (4), normalization process theory (e.g., May 2013), health systems frameworks, notably the Integrated Promoting Action on Research Implementation in Health Services (7), and complexity science (25-26). Berta et al (2015) (4) applies organizational learning theory to describe how healthcare facilitation supports evolving understanding of organizational processes at higher levels than day-to-day job duties among healthcare organization employees (e.g., sense-making, or systems thinking). May’s Normalization Process Theory, for example, suggests that healthcare facilitation may work through increases in individual and collective commitment, that in turn increase the organizational capacity to cooperate and coordinate actions through changes in role definition, knowledge and norms, and emergent expressions of agency that include collective action and cognitive participation (27).

The iPARIHS (Integrated Promoting Action on Research Implementation in Health Services) framework (7) is a health systems framework that has been increasingly used to explain how healthcare facilitation works to effect practice change. Deriving its origins from the behavior change, organizational psychology, and public health literature, iPARIHS describes healthcare facilitation as working to identify and address barriers and facilitators to innovation uptake. iPARIHS also includes organizational contextual factors such as health systems that are thought to influence healthcare facilitation effectiveness and innovation update. Through iPARIHS and May’s Normalization Process Theory, specific mechanisms through which healthcare facilitation promotes practice change have been hypothesized to include the building of supportive interpersonal relationships in which the facilitator’s role is accepted by the practice and enables them to identify and prepare champions of innovation, garner leadership support, and further motivate the integration of innovation into practice (4, 7).

Complexity science presents healthcare facilitation as a non-linear, complex adaptive process. It can describe how core leadership (e.g., listening, reflection) and management (e.g., adaptability, resilience) characteristics interact to explain practice change, which in some cases may not always be predictable (18-19, 25-26). Complexity science also considers that healthcare facilitators strive to develop relationships and build trust over time with organization members, by engaging in meaningful conversations to foster continuous learning, that in turn shape (and are shaped) by the organization members’ motivations (28). Healthcare facilitators also work with employees to expand opportunities for practice change and support the mobilization of resources to improve quality (29). Facilitators who understand healthcare organizations as complex adaptive systems use a relationship-based quality improvement approach to help practices facing uncertainty make change (25). For example, healthcare facilitators recognizing these “malleable moments” can successfully leverage an organization’s openness to change (18).

These theories and frameworks build the necessary background to help identify, evaluate, and test hypotheses regarding healthcare facilitation components and mechanisms. They are also essential to understanding the context in which healthcare facilitators operate and therefore can impact effectiveness across different settings. For example, organizational capacity (30-31) or readiness (31-32) can influence healthcare facilitation success (23, 34). Some studies have also examined which organizational moderators (e.g., culture, climate) as well as practitioner or clinical team-level mediators (e.g., self-efficacy, burnout, turnover) were most conducive to successful healthcare facilitation (33). In some studies, sites that had a more entrepreneurial culture benefitted most from healthcare facilitation (35-36). Healthcare facilitation effectiveness can also be impacted by sites’ perceptions of leadership priorities, staff shortages, and turnover (35). In low- and middle-income countries (LMICs), contextual factors are often influenced by a hierarchical culture as well as whether the healthcare system is financed by donors or the government (37-38). Moreover, in LMICs as well as in large national health systems, the effects of healthcare facilitation in LMICs might be confounded if offered as part of a larger quality improvement rollout or as a top-down mandate (39-40).

**Recent evidence of healthcare facilitation’s effectiveness**

Since the publication of the Berta et al. (2015) (4) landmark paper, there has been more empirical studies in the U.S. and elsewhere assessing the effectiveness of healthcare facilitation in improving effective innovation uptake and patient outcomes. Many of these studies used cluster randomized trial designs involving site-level randomization and assessed whether healthcare facilitation compared to lower-intensity strategies such as manual dissemination and training improved uptake of effective innovations, including preventive services (41), behavioral health care (3, 23-24), and chronic disease management (6, 42). Although found to be effective in improving effective innovation uptake and/or patient outcomes, there was variation across these studies in level of effectiveness across different settings including larger health systems and smaller practices in the U.S. and LMICs. Some of these studies, provided in more detail below, assessed components and mechanisms in greater detail to inform the mechanism mapping process.

Recent evidence identifying healthcare facilitation components

In a systematic review and meta-analysis of over 20 mostly U.S. based studies (43) healthcare facilitation was associated with improved effective innovation uptake compared to usual dissemination practices in primary care settings. In these studies, healthcare facilitation was usually delivered by an outside expert who had regular contacts with healthcare organization employees and sometimes with the managers and leaders of the clinics’ larger organization. Healthcare facilitators have been most effective when they worked with clinicians at the local site, usually via in-person meetings (3, 6, 19, 44). However, healthcare facilitation has been delivered effectively virtually as well (23,24,34), especially when healthcare facilitators first met with practitioners in-person (34). An evidence review conducted in 2017 of implementation strategies used to scale up effective innovations (45) also unpacked key components of healthcare facilitation that helped achieve its success included skills development in influencing (e.g., engaging relevant leadership, identifying and preparing champions), problem-solving (e.g., interactive discussions to identify organizational capacities and opportunities to promote effective innovation adoption), and coalition-building (e.g., allowing flexibility to incorporate practice insights that help with motivation).

Empirical evidence related to healthcare facilitation since these evidence reviews from lower resourced U.S. practices and LMICs also identifies key healthcare facilitation core components in enhancing the uptake of effective innovations, but with mixed results for patient outcomes. In a national randomized adaptive trial of virtual healthcare facilitation added to a low-intensity implementation strategy (Replicating Effective Programs-REP: manual dissemination, training, group-based technical assistance), providers at Veterans Health Administration (VHA) facilities in the U.S. receiving virtual healthcare facilitation to REP improved uptake of a brief care management program for persons with serious mental illness but had no effect on patient outcomes (24). The virtual healthcare facilitation consisted of 6 months of consultation provided by the facilitator to the practitioner implementing the program and consisted of topics such as problem-solving barriers to uptake, using quality improvement processes to adapt the program to overcome local barriers, and communicating the value of the program to leadership. In smaller primary care practices. In another multisite randomized trial, Parchman et al (2019) found that in-person healthcare facilitation plus additional educational outreach and shared learning opportunities among staff were associated with improved patient achievement of blood pressure goals compared to practices randomized to receive healthcare facilitation alone (6).

Among LMICs, most studies have been observational in nature and usually involved donor-funded initiatives. One study in Vietnam that applied healthcare facilitation to improve HIV care showed successful uptake, integration, and sustainment of quality improvement within provincial and district level health services nationally (46). In Brazil, healthcare facilitation was associated with improved maternal and newborn care practices across six maternity hospitals but with marked heterogeneity in outcomes per World Health Organization metrics (47). Zamboni, Singh, Tyagi, et al (2021) (38) reported poor outcomes in the Safe Care Saving Lives program in India where external healthcare facilitators worked with local teams to improve maternal and newborn care practices in 29 networked hospitals, citing poor leadership engagement and hierarchical relationships between staff as barriers to successful implementation.

Current evidence of specific healthcare facilitation mechanisms

Recent studies have also begun to identify potential mechanisms of healthcare facilitation. For example, in the recently completed Evaluating System Change to Advance Learning and Take Evidence to Scale (ESCALATES) study (48), 160 healthcare facilitators worked with 1600 small primary care practices to implement the EvidenceNOW cardiovascular disease (CVD) care initiative. Overall, sites with the most improvement in CVD care had healthcare facilitators who excelled at cultivating motivation by adapting quality improvement processes, addressing practice resistance, guiding clinical teams to think critically, and providing accountability to support change (44). These proposed mechanisms were independent of the background or experience of the healthcare facilitator (49). Similarly, in the Adaptive Implementation of Effective Programs Trial (ADEPT), healthcare facilitators working with over 50 community-based mental health outpatient settings improved uptake of a collaborative care model by teaching practitioners strategic thinking skills such as how to align program successes with leadership priorities and motivating and inspiring other employees to become champions (23,34). These core facilitation activities and mechanisms have also been identified based on extensive work by the Behavioral Health Quality Enhancement Research Initiative. (50-51).

Overall, the literature suggests that healthcare facilitation can effectively improve implementation of effective innovations. However, there is also significant variation in outcomes due to lack of clear definitions of its components and mechanisms. A concerted effort to better define healthcare facilitation components and their mechanisms more clearly will enhance its impact in U.S. as well as LMIC settings where an increasing number of implementation studies are being conducted. The table below presents a summary of healthcare facilitation components based on this literature review that informed the mechanism mapping process described in this paper.

**Table. Consolidated Description of Healthcare Facilitation Components: Proctor Framework**

| The Actor(s) | Facilitator meets with practice manager and practitioners (e.g., clinicians and other employees) responsible for implementing effective innovation |
| --- | --- |
| The Action(s) | Build supportive relationships   - Assess for readiness and identify barriers - Organize implementation teams - Openness to change as driven by perceived vision and values   Problem solving activities   - Motivate and inspire employees/clinicians in using the effective innovation - Create measurable goals, specify tasks/timeline using audit and feedback - Map out workflows and evaluate staff roles - Identify and prepare additional site champions - Coalition-build interrelationships across staff, leaders to address barriers - Connect and discuss issues with similar sites - Ongoing training where needed   Ongoing monitoring   - Audit and feedback using tailored case review and skill development - Local technical assistance via ongoing consultation on coordination of care   Plan for sustainment   - Conduct educational outreach meetings in practice - Promote to leadership positive recognition and support - Develop business plan for sustainability, e.g., reimbursement models |
| Action Targets | Clinicians, employees, and managers (clinical teams)   - Embed clinic evidence into daily work - Use data and information technology to understand and improve care - Establish a regular quality improvement (QI) process - Identify at-risk patients for outreach - Define roles and responsibilities for improving care - Deepen patient self-management support - Link patients to resources outside of the clinic |
| Temporality | Regular meetings for at least 6 months, ideally at least 12-18 months |
| Dose | Weekly or biweekly meetings usually ~1 hour each, timing can be tapered over time |
| Implementation outcome affected | - Behavior change to promote effective innovation uptake - Fidelity to the effective innovation as delivered by healthcare practitioners - Workflow maps - Revisions to care team roles and responsibilities - Modified staff routines to enhance efficiency |

**References**

1. Proctor EK, Powell BJ, McMillen JC. Implementation strategies: recommendations for specifying and reporting. Implement Sci. 2013 Dec 1;8:139. doi: 10.1186/1748-5908-8-139. PMID: 24289295; PMCID: PMC3882890.

2. Powell BJ, Waltz TJ, Chinman MJ, Damschroder LJ, Smith JL, Matthieu MM, Proctor EK, Kirchner JE. A refined compilation of implementation strategies: results from the Expert Recommendations for Implementing Change (ERIC) project. Implement Sci. 2015 Feb 12; 10:21. doi: 10.1186/s13012-015-0209-1.

3. Kirchner JE, Ritchie MJ, Pitcock JA, Parker LE, Curran GM, Fortney JC. Outcomes of a partnered facilitation strategy to implement primary care-mental health. J Gen Intern Med. 2014 Dec;29 Suppl 4(Suppl 4):904-12. doi: 10.1007/s11606-014-3027-2. PMID: 25355087; PMCID: PMC4239280.

4. Berta W, Cranley L, Dearing JW, Dogherty EJ, Squires JE, Estabrooks CA. Why (we think) facilitation works: insights from organizational learning theory. Implement Sci. 2015 Oct 6;10:141. doi: 10.1186/s13012-015-0323-0. PMID: 26443999; PMCID: PMC4596304.

5. Stetler CB, Legro MW, Rycroft-Malone J, Bowman C, Curran G, Guihan M, Hagedorn H, Pineros S, Wallace CM. Role of "external facilitation" in implementation of research findings: a qualitative evaluation of facilitation experiences in the Veterans Health Administration. Implement Sci. 2006 Oct 18;1:23. doi: 10.1186/1748-5908-1-23. PMID: 17049080; PMCID: PMC1635058.

6. Parchman ML, Anderson ML, Dorr DA, Fagnan LJ, O'Meara ES, Tuzzio L, Penfold RB, Cook AJ, Hummel J, Conway C, Cholan R, Baldwin LM. A Randomized Trial of External Practice Support to Improve Cardiovascular Risk Factors in Primary Care. Ann Fam Med. 2019 Aug 12;17(Suppl 1):S40-S49. doi: 10.1370/afm.2407. PMID: 31405875; PMCID: PMC6827661.

7. Harvey G, Kitson A. PARIHS revisited: from heuristic to integrated framework for the successful implementation of knowledge into practice. Implement Sci. 2016 Mar 10;11:33. doi: 10.1186/s13012-016-0398-2. PMID: 27013464; PMCID: PMC4807546.

8. Kitson A, Harvey G, McCormack B. Enabling the implementation of evidence-based practice: a conceptual framework. Qual Health Care. 1998 Sep;7(3):149-58. doi: 10.1136/qshc.7.3.149. PMID: 10185141; PMCID: PMC2483604.

9. Seers K, Rycroft-Malone J, Cox K, Crichton N, Edwards RT, Eldh AC, Estabrooks CA, Harvey G, Hawkes C, Jones C, Kitson A, McCormack B, McMullan C, Mockford C, Niessen T, Slater P, Titchen A, van der Zijpp T, Wallin L. Facilitating Implementation of Research Evidence (FIRE): an international cluster randomised controlled trial to evaluate two models of facilitation informed by the Promoting Action on Research Implementation in Health Services (PARIHS) framework. Implement Sci. 2018 Nov 16;13(1):137. doi: 10.1186/s13012-018-0831-9. PMID: 30442174; PMCID: PMC6238407.

10. Harvey G, McCormack B, Kitson A, Lynch E, Titchen A. Designing and implementing two facilitation interventions within the 'Facilitating Implementation of Research Evidence (FIRE) study: a qualitative analysis from an external facilitators' perspective. Implement Sci. 2018 Nov 16;13(1):141. doi: 10.1186/s13012-018-0812-z. PMID: 30442157; PMCID: PMC6238352.

11. Kilbourne AM, Neumann MS, Pincus HA, Bauer MS, Stall R. Implementing evidence-based interventions in health care: application of the replicating effective programs framework. Implement Sci. 2007 Dec 9;2:42. doi: 10.1186/1748-5908-2-42. PMID: 18067681; PMCID: PMC2248206.

12. Ritchie MJ, Parker LE, Kirchner JE. From novice to expert: a qualitative study of implementation facilitation skills. Implement Sci Commun. 2020 Feb 25;1:25. doi: 10.1186/s43058-020-00006-8. PMID: 32885184; PMCID: PMC7427882.

13. Kilbourne AM, Goodrich DE, Miake-Lye I, Braganza MZ, Bowersox NW. Quality Enhancement Research Initiative Implementation Roadmap: Toward Sustainability of Evidence-based Practices in a Learning Health System. Med Care. 2019 Oct;57 Suppl 10 Suppl 3(10 Suppl 3):S286-S293. doi: 10.1097/MLR.0000000000001144. PMID: 31517801; PMCID: PMC6750196.

14. Walunas TL, Ye J, Bannon J, Wang A, Kho AN, Smith JD, Soulakis N. Does coaching matter? Examining the impact of specific practice facilitation strategies on implementation of quality improvement interventions in the Healthy Hearts in the Heartland study. Implement Sci. 2021 Mar 31;16(1):33. doi: 10.1186/s13012-021-01100-8. PMID: 33789696; PMCID: PMC8011080.

15. Jordan ME, Lanham HJ, Crabtree BF, Nutting PA, Miller WL, Stange KC, McDaniel RR Jr. The role of conversation in health care interventions: enabling sensemaking and learning. Implement Sci. 2009 Mar 13;4:15. doi: 10.1186/1748-5908-4-15. PMID: 19284660; PMCID: PMC2663543.

16. Ritchie MJ, Parker LE, Edlund CN, Kirchner JE. Using implementation facilitation to foster clinical practice quality and adherence to evidence in challenged settings: a qualitative study. BMC Health Serv Res. 2017 Apr 20;17(1):294. doi: 10.1186/s12913-017-2217-0. PMID: 28424052; PMCID: PMC5397744.

17. Rycroft-Malone J, Seers K, Eldh AC, Cox K, Crichton N, Harvey G, Hawkes C, Kitson A, McCormack B, McMullan C, Mockford C, Niessen T, Slater P, Titchen A, van der Zijpp T, Wallin L. A realist process evaluation within the Facilitating Implementation of Research Evidence (FIRE) cluster randomised controlled international trial: an exemplar. Implement Sci. 2018 Nov 16;13(1):138. doi: 10.1186/s13012-018-0811-0. PMID: 30442165; PMCID: PMC6238283.

18. Ruhe MC, Weyer SM, Zronek S, Wilkinson A, Wilkinson PS, Stange KC. Facilitating practice change: lessons from the STEP-UP clinical trial. Prev Med. 2005 Jun;40(6):729-34. doi: 10.1016/j.ypmed.2004.09.015. PMID: 15850872.

19. Cohen DJ, Balasubramanian BA, Lindner S, Miller WL, Sweeney SM, Hall JD, Ward R, Marino M, Springer R, McConnell KJ, Hemler JR, Ono SS, Ezekiel-Herrera D, Baron A, Crabtree BF, Solberg LI. How Does Prior Experience Pay Off in Large-Scale Quality Improvement Initiatives? J Am Board Fam Med. 2022 Sep 16:jabfm.2022.AP.220088. doi: 10.3122/jabfm.2022.AP.220088. Epub ahead of print. PMID: 36113993.

20. Smith SN, Almirall D, Choi SY, Koschmann E, Rusch A, Bilek E, Lane A, Abelson JL, Eisenberg D, Himle JA, Fitzgerald KD, Liebrecht C, Kilbourne AM. Primary aim results of a clustered SMART for developing a school-level, adaptive implementation strategy to support CBT delivery at high schools in Michigan. Implement Sci. 2022 Jul 8;17(1):42. doi: 10.1186/s13012-022-01211-w. Erratum in: Implement Sci. 2022 Aug 11;17(1):54. PMID: 35804370; PMCID: PMC9264291.

21. Kolko DJ, McGuier EA, Turchi R, Thompson E, Iyengar S, Smith SN, Hoagwood K, Liebrecht C, Bennett IM, Powell BJ, Kelleher K, Silva M, Kilbourne AM. Care team and practice-level implementation strategies to optimize pediatric collaborative care: study protocol for a cluster-randomized hybrid type III trial. Implement Sci. 2022 Feb 22;17(1):20. doi: 10.1186/s13012-022-01195-7. PMID: 35193619; PMCID: PMC8862323.

22. Eisman AB, Hutton DW, Prosser LA, Smith SN, Kilbourne AM. Cost-effectiveness of the Adaptive Implementation of Effective Programs Trial (ADEPT): approaches to adopting implementation strategies. Implement Sci. 2020 Dec 14;15(1):109. doi: 10.1186/s13012-020-01069-w. PMID: 33317593; PMCID: PMC7734829.

23. Smith SN, Almirall D, Prenovost K, Liebrecht C, Kyle J, Eisenberg D, Bauer MS, Kilbourne AM. Change in Patient Outcomes After Augmenting a Low-level Implementation Strategy in Community Practices That Are Slow to Adopt a Collaborative Chronic Care Model: A Cluster Randomized Implementation Trial. Med Care. 2019 Jul;57(7):503-511. doi: 10.1097/MLR.0000000000001138. PMID: 31135692; PMCID: PMC6684247.

24. Kilbourne AM, Almirall D, Goodrich DE, Lai Z, Abraham KM, Nord KM, Bowersox NW. Enhancing outreach for persons with serious mental illness: 12-month results from a cluster randomized trial of an adaptive implementation strategy. Implement Sci. 2014 Dec 28;9:163. doi: 10.1186/s13012-014-0163-3. PMID: 25544027; PMCID: PMC4296543.

26. May C. Towards a general theory of implementation. Implement Sci. 2013 Feb 13;8:18. doi: 10.1186/1748-5908-8-18. PMID: 23406398; PMCID: PMC3602092.

26. Leykum LK, Lanham HJ, Pugh JA, Parchman M, Anderson RA, Crabtree BF, Nutting PA, Miller WL, Stange KC, McDaniel RR. Manifestations and implications of uncertainty for improving healthcare systems: an analysis of observational and interventional studies grounded in complexity science. Implement Sci. 2014 Nov 19;9:165. doi: 10.1186/s13012-014-0165-1. PMID: 25407138; PMCID: PMC4239371.

27. McDaniel RR Jr, Driebe DJ, Lanham HJ. Health care organizations as complex systems: new perspectives on design and management. Adv Health Care Manag. 2013;15:3-26. doi: 10.1108/s1474-8231(2013)0000015007. PMID: 24749211.

28. Crabtree BF, Nutting PA, Miller WL, McDaniel RR, Stange KC, Jaen CR, Stewart E. Primary care practice transformation is hard work: insights from a 15-year developmental program of research. Med Care. 2011 Dec;49 Suppl(Suppl):S28-35. doi: 10.1097/MLR.0b013e3181cad65c. PMID: 20856145; PMCID: PMC3043156.

29. Cohen D, McDaniel RR Jr, Crabtree BF, Ruhe MC, Weyer SM, Tallia A, Miller WL, Goodwin MA, Nutting P, Solberg LI, Zyzanski SJ, Jaén CR, Gilchrist V, Stange KC. A practice change model for quality improvement in primary care practice. J Healthc Manag. 2004 May-Jun;49(3):155-68; discussion 169-70. PMID: 15190858.

30. Allen JD, Towne SD Jr, Maxwell AE, DiMartino L, Leyva B, Bowen DJ, Linnan L, Weiner BJ. Meausures of organizational characteristics associated with adoption and/or implementation of innovations: A systematic review. BMC Health Serv Res. 2017 Aug 23;17(1):591. doi: 10.1186/s12913-017-2459-x. PMID: 28835273; PMCID: PMC5569532.

31. Nguyen AM, Cuthel A, Padgett DK, Niles P, Rogers E, Pham-Singer H, Ferran D, Kaplan SA, Berry C, Shelley D. How Practice Facilitation Strategies Differ by Practice Context. J Gen Intern Med. 2020 Mar;35(3):824-831. doi: 10.1007/s11606-019-05350-7. Epub 2019 Oct 21. PMID: 31637651; PMCID: PMC7080927.

32. Helfrich CD, Li YF, Sharp ND, Sales AE. Organizational readiness to change assessment (ORCA): development of an instrument based on the Promoting Action on Research in Health Services (PARIHS) framework. Implement Sci. 2009 Jul 14;4:38. doi: 10.1186/1748-5908-4-38. PMID: 19594942; PMCID: PMC2716295.

33. Connolly SL, Sullivan JL, Ritchie MJ, Kim B, Miller CJ, Bauer MS. External facilitators' perceptions of internal facilitation skills during implementation of collaborative care for mental health teams: a qualitative analysis informed by the i-PARIHS framework. BMC Health Serv Res. 2020 Mar 4;20(1):165. doi: 10.1186/s12913-020-5011-3. PMID: 32131824; PMCID: PMC7057643.

34. Smith SN, Liebrecht CM, Bauer MS, Kilbourne AM. Comparative effectiveness of external vs blended facilitation on collaborative care model implementation in slow-implementer community practices. Health Serv Res. 2020 Dec;55(6):954-965. doi: 10.1111/1475-6773.13583. Epub 2020 Oct 30. PMID: 33125166; PMCID: PMC7704469.

35. Smith SN, Almirall D, Prenovost K, Goodrich DE, Abraham KM, Liebrecht C, Kilbourne AM. Organizational culture and climate as moderators of enhanced outreach for persons with serious mental illness: results from a cluster-randomized trial of adaptive implementation strategies. Implement Sci. 2018 Jul 9;13(1):93. doi: 10.1186/s13012-018-0787-9. PMID: 29986765; PMCID: PMC6038326.

36. Rogers ES, Cuthel AM, Berry CA, Kaplan SA, Shelley DR. Clinician Perspectives on the Benefits of Practice Facilitation for Small Primary Care Practices. Ann Fam Med. 2019 Aug 12;17(Suppl 1):S17-S23. doi: 10.1370/afm.2427. PMID: 31405872; PMCID: PMC6827665.

37. Ikeda DJ, Basenero A, Murungu J, Jasmin M, Inimah M, Agins BD. Implementing quality improvement in tuberculosis programming: Lessons learned from the global HIV response. J Clin Tuberc Other Mycobact Dis. 2019 Aug 7;17:100116. doi: 10.1016/j.jctube.2019.100116. PMID: 31788558; PMCID: PMC6879975.

38. Zamboni K, Singh S, Tyagi M, Hill Z, Hanson C, Schellenberg J. Effect of collaborative quality improvement on stillbirths, neonatal mortality and newborn care practices in hospitals of Telangana and Andhra Pradesh, India: evidence from a quasi-experimental mixed-methods study. Implement Sci. 2021 Jan 7;16(1):4. doi: 10.1186/s13012-020-01058-z. PMID: 33413504; PMCID: PMC7788546.

39. Zaka N, Alexander EC, Manikam L, Norman ICF, Akhbari M, Moxon S, Ram PK, Murphy G, English M, Niermeyer S, Pearson L. Quality improvement initiatives for hospitalised small and sick newborns in low- and middle-income countries: a systematic review. Implement Sci. 2018 Jan 25;13(1):20. doi: 10.1186/s13012-018-0712-2. PMID: 29370845; PMCID: PMC5784730.

40. Hargreaves JR, Hassan S, Schellenberg J, Hayes R, Webster J, Lewis JJ. Five Challenges in the Design and Conduct of IS Trials for HIV Prevention and Treatment. J Acquir Immune Defic Syndr. 2019 Dec;82 Suppl 3:S261-S270. doi: 10.1097/QAI.0000000000002192. PMID: 31764262.

41. Gold R, Bunce A, Cowburn S, Davis JV, Nelson JC, Nelson CA, Hicks E, Cohen DJ, Horberg MA, Melgar G, Dearing JW, Seabrook J, Mossman N, Bulkley J. Does increased implementation support improve community clinics' guideline-concordant care? Results of a mixed methods, pragmatic comparative effectiveness trial. Implement Sci. 2019 Dec 5;14(1):100. doi: 10.1186/s13012-019-0948-5. PMID: 31805968; PMCID: PMC6894475.

42. Perry CK, Damschroder LJ, Hemler JR, Woodson TT, Ono SS, Cohen DJ. Specifying and comparing implementation strategies across seven large implementation interventions: a practical application of theory. Implement Sci. 2019 Mar 21;14(1):32. doi: 10.1186/s13012-019-0876-4. PMID: 30898133; PMCID: PMC6429753.

43. Baskerville NB, Liddy C, Hogg W. Systematic review and meta-analysis of practice facilitation within primary care settings. Ann Fam Med. 2012 Jan-Feb;10(1):63-74. doi: 10.1370/afm.1312. PMID: 22230833; PMCID: PMC3262473.

44. Sweeney SM, Hemler JR, Baron AN, Woodson TT, Ono SS, Gordon L, Crabtree BF, Cohen DJ. Dedicated Workforce Required to Support Large-Scale Practice Improvement. J Am Board Fam Med. 2020 Mar-Apr;33(2):230-239. doi: 10.3122/jabfm.2020.02.190261. PMID: 32179606; PMCID: PMC7175633.

45. Miake-Lye I, Mak S, Lam CA, Lambert-Kerzner AC, Delevan D, Olmos-Ochoa T, Shekelle P. Scaling Beyond Early Adopters: A Content Analysis of Literature and Key Informant Perspectives. J Gen Intern Med. 2021 Feb;36(2):383-395. doi: 10.1007/s11606-020-06142-0. Epub 2020 Oct 27. PMID: 33111242; PMCID: PMC7878615.

46. Cosimi LA, Dam HV, Nguyen TQ, Ho HT, Do PT, Duc DN, Nguyen HT, Gardner B, Libman H, Pollack T, Hirschhorn LR. Integrated clinical and quality improvement coaching in Son La Province, Vietnam: a model of building public sector capacity for sustainable HIV care delivery. BMC Health Serv Res. 2015 Jul 17;15:269. doi: 10.1186/s12913-015-0935-8. PMID: 26184505; PMCID: PMC4504451.

47. Tamburlini G, Bacci A, Daniele M, Hodorogea S, Jeckaite D, Maciulevicius A, Valente EP, Siupsinskas G, Uxa F, Vezzini F, Lincetto O, Bucagu M. Use of a participatory quality assessment and improvement tool for maternal and neonatal hospital care. Part 2: Review of the results of quality cycles and of factors influencing change. J Glob Health. 2020 Dec;10(2):020433. doi: 10.7189/jogh.10.020433. PMID: 33403105; PMCID: PMC7750017.

48. Cohen DJ, Balasubramanian BA, Gordon L, Marino M, Ono S, Solberg LI, Crabtree BF, Stange KC, Davis M, Miller WL, Damschroder LJ, McConnell KJ, Creswell J. A national evaluation of a dissemination and implementation initiative to enhance primary care practice capacity and improve cardiovascular disease care: the ESCALATES study protocol. Implement Sci. 2016 Jun 29;11(1):86. doi: 10.1186/s13012-016-0449-8. PMID: 27358078; PMCID: PMC4928346.

49. Sweeney SM, Baron A, Hall JD, Ezekiel-Herrera D, Springer R, Ward RL, Marino M, Balasubramanian BA, Cohen DJ. Effective Facilitator Strategies for Supporting Primary Care Practice Change: A Mixed Methods Study. Annals of Family Medicine Sep 2022, 20 (5) 414-422; DOI: 10.1370/afm.2847

50. Ritchie MJ, Drummond KL, Smith BN, Sullivan JL, Landes SJ. Development of a qualitative data analysis codebook informed by the i-PARIHS framework. Implement Sci Commun. 2022 Sep 14;3(1):98. doi: 10.1186/s43058-022-00344-9. PMID: 36104801; PMCID: PMC9476709.

51. Smith JL, Ritchie MJ, Kim B, Miller CJ, Chinman MJ, Kelly PA, Landes SJ, Kirchner JE. Getting to fidelity: Scoping review and expert panel process to identify core activities of implementation facilitation strategies. Available at: <https://osf.io/preprints/socarxiv/6xfvj/>
